# The National Specialists’ Associations

**Published:** 1890-07

**Authors:** 


THE national specialist associations are covering themselves with anything but glory in the methods adopted to defeat candidates for membership. Some years ago a physician was proposed for membership in the American Neurological Association who scientifically bore a high reputation. His sponsors were two leading American neurologists. His thesis was approved by one of the foremost American cerebral anatomists, but a small New York family clique tried to defeat the candidate in committee. The association soon demolished such star-chamber proceedings and elected the candidate. The American Genito-Urinary Surgeons Association refused membership to a leading American syphilographer, on a pseudonymous letter from a Chicago surgeon, now under suspicion for most dastardly secret unprofessional slanders against physicians, who has been censured for unprofessional conduct by the Chicago Medical society. The American Surgical Association seems to be following in the wake of the organization just named, for it has rejected an admittedly unobjectionable candidate at the instance of the horse-marine ex-editor of the *Journal of the American Medical Association.* If these national organizations continue to indulge in such puerile performances they will soon degenerate into coteries of mutual admirationists and the really scientific specialists will be forced to find a congenial home in sections of the American Medical Association.—*Chicago Medical Standard, June.*

